# Supplementary material for: Long-Term Outcomes and Causes of Death among Medullary Thyroid Carcinoma Patients with Distant Metastases
Source: Cancers (Basel). 2021 Sep 17;13(18):4670. doi: 10.3390/cancers13184670 (PMC8469864; doi:10.3390/cancers13184670)
Supplement: Supplementary file 1 [file cancers-13-04670-s001.zip › cancers-1337598-supplementary.pdf]

cancers

MDPI

Supplementary Files

Long-Term Outcomes and Causes of Death among Medullary Thyroid Carcinoma Patients with Distant Metastases

Hyunju Park, Heera Yang, Jung Heo, Tae Hyuk Kim, Sun Wook Kim and Jae Hoon Chung

Table S1. Prognostic factors for cancer specific survival in patients with medullary thyroid cancer and distant metastasis.

**Characteristics**	**Unadjusted**		**Adjusted**	
	**HR (95% CI)**	***p* value**	**HR (95% CI)**	***p* value**
Age, years	1.04 (1.01–1.08)	0.016		
Sex				
male	Reference			
female	0.48 (0.18–1.27)	0.140		
Primary tumor size, cm				
≤ 2.0	Reference			
> 2.0 and ≤ 4.0	4.63 (0.95–22.45)	0.057		
> 4.0	5.27 (1.09–25.49)	0.039		
Cervical lymph node metastasis				
No	Reference			
Yes	0.495 (0.18–1.40)	0.184		
Initial distant metastasis				
No	Reference		Reference	
Yes	7.41 (2.55–21.53)	<0.001	18.89 (4.35–82.11)	<0.001
Initial distant metastasis site				
Lung	Reference		Reference	
Mediastinum	0.98 (0.19–5.10)	0.984	0.80 (0.14–4.52)	0.801
Bone	3.01 (0.59–15.44)	0.187	12.66 (1.62–98.98)	0.016
Liver	-	-	-	-
Multiple sites	6.16 (1.64–23.14)	0.007	7.17 (1.80–28.52)	0.005

HR; hazard ratio, CI; confidential interval.
